# Nomegestrol Acetate Suppresses Human Endometrial Cancer RL95-2 Cells Proliferation In Vitro and In Vivo Possibly Related to Upregulating Expression of SUFU and Wnt7a

**DOI:** 10.3390/ijms18071337

**Published:** 2017-06-22

**Authors:** A-ying Ma, Shu-wu Xie, Jie-yun Zhou, Yan Zhu

**Affiliations:** Lab of Reproductive Pharmacology, Key Lab of Reproduction Regulation of NPFPC, SIPPR, IRD, Fudan University, Shanghai 200032, China; ayma14@fudan.edu.cn (A.-y.M.); xieshw@fudan.edu.cn (S.-w.X.); zjy_sans@sippr.org (J.-y.Z.)

**Keywords:** nomegestrol acetate, endometrial cancer, RL95-2 cells, KLE cells, proliferation, SUFU, Wnt7a

## Abstract

Nomegestrol acetate (NOMAC) has been successfully used for the treatment of some gynecological disorders, and as a combined oral contraceptive with approval in many countries. In this study, we investigated the effects of NOMAC on human endometrial cancer cells in vitro and in vivo. The proliferation of human endometrial cancer cells (RL95-2 and KLE) were assessed using CCK-8 and EdU incorporation assays. Whole-genome cDNA microarray analysis was used to identify the effects of NOMAC on gene expression profiles in RL95-2 cells. RL95-2 xenograft nude mice were treated with NOMAC (50, 100, and 200 mg/kg) or medroxyprogesterone acetate (MPA; 100 and 200 mg/kg) for 28 consecutive days. The results showed that NOMAC significantly inhibited the growth of RL95-2 cells in a concentration-dependent manner, but not in KLE cells. Further investigation demonstrated that NOMAC produced a stronger inhibition of tumor growth (inhibition rates for 50, 100, and 200 mg/kg NOMAC were 24.74%, 47.04%, and 58.06%, respectively) than did MPA (inhibition rates for 100 and 200 mg/kg MPA were 41.06% and 27.01%, respectively) in the nude mice bearing the cell line of RL95-2. NOMAC altered the expression of several genes related to cancer cell proliferation, including *SUFU* and *Wnt7a*. The upregulation of *SUFU* and *Wnt7a* was confirmed using real-time quantitative polymerase chain reaction and Western blotting in RL95-2 cells and RL95-2 xenograft tumor tissues, but not in KLE cells. These data indicate that NOMAC can inhibit the proliferation of RL95-2 cell in vitro and suppress the growth of xenografts in the nude mice bearing the cell line of RL95-2 in vivo. This effect could be related to the upregulating expression of SUFU and Wnt7a.

## 1. Introduction

Endometrial cancer is one of the most common gynecological malignancies, and its incidence has markedly increased around the world, especially in developed countries. Endometrioid adenocarcinoma accounts for over 80% of all endometrial cancers, and approximately 5–14% of affected women are younger than 40 years [1,2]. When arising in women of childbearing age, endometrial cancer usually presents with favorable prognostic features, including an endometrioid hystotype, a well-differentiated lesion, or/and an absent or minimal myometrial invasion, which belongs to type I endometrial cancer (estrogen/progesterone receptor (ER/PR) positive) [3,4]. The standard treatment for type I endometrial cancer is surgery and adjuvant therapy (e.g., progestin, radiotherapy, and chemotherapy) [5]. Although hysterectomy with bilateral oophorectomy usually leads to complete remission and long-term survival, this intervention eliminates any potential for fertility [6]. Therefore, more efficacious and safer medical therapies are required to improve the management of type I endometrial cancer, especially for patients who are suffering endometrioid adenocarcinoma and wish to preserve fertility.

Endometrial cancer is a hormone-regulated cancer, estrogen drives its growth, and progesterone suppresses its proliferation and leads to differentiation [7]. Thus, various derivatives of progesterone have been used to treat this type of cancer [8], such as medroxyprogesterone acetate (MPA) and megestrol acetate (MA) [9,10,11,12]. However, MPA is able to induce the development of thrombosis [13] and must be discontinued if a clotting abnormality is detected. This adverse effect of MPA can be fatal through inducing cerebral infarction, myocardial infarction, or pulmonary embolism. There have also been many cases of ineffectiveness or recurrence. Therefore, new progestogen with greater efficacy and fewer side effects are desirable.

The fourth-generation progestogen nomegestrol acetate (NOMAC) is a potent, highly selective progestogen with higher progesterone activity than MPA [14], and has been used successfully to treat hormone-dependent gynecological disorders (menstrual disturbances, dysmenorrhea, and premenstrual syndrome) [15] and as a component of hormone replacement therapy in combination with estradiol for the relief of menopausal symptoms [16]. In a combined oral contraceptive, NOMAC combined with estradiol achieved effective contraception with good cycle control and a favorable safety profile. NOMAC does not increase the risk of suffering breast cancer. Therefore, when NOMAC is used to treat endometrial cancer in the future, we anticipate that NOMAC will not increase the risk of breast cancer, and might inhibit breast cancer [17,18,19]. NOMAC is a 19-norprogesterone derivative designed to bind specifically to the progesterone receptor, and is relatively lacking in affinity to other steroid receptors [20,21]. NOMAC exerts strong antiestrogenic effects at the level of the endometrium and has potent antigonadotropic activity, but without any residual androgenic or glucocorticoid properties [22]. Therefore, NOMAC greatly reduces major side effects, such as water retention, and due to its antiandrogenic properties, it eliminates all forms of hyperandrogenism associated with acne and mild hirsutism [23,24,25]. As far as our knowledge, its anticancer activity has not been reported, and the mechanism of action of NOMAC is yet to be thoroughly discovered.

The Hedgehog (Hh) signaling pathway plays numerous roles in the control of cell proliferation and survival, tissue patterning and stem cell maintenance. The suppressor of fused homolog gene (*SUFU*), a Hh pathway inhibitor at a pivotal site, has been confirmed to affect the occurrence and development of tumors [26], since SUFU functions in the mammalian Hh signaling pathway by either impeding the nuclear localization of Gli proteins or acting as a transcription corepressor of GLIs [26,27]. Further, a study reported that the Hh signaling pathway regulates the Wnt signaling pathway by upregulating secreted frizzled-related protein 1 (*SFRP1*) in interstitial cells [28]. Wnt/β-catenin pathway plays an important role in the development of endometrial cancer. Approximately 40% of endometrial cancer shows abnormal activation of the Wnt/β-catenin pathway, in which most of them belongs to the type I endometrial cancer [29,30]. β-Catenin is a hallmark of the activated Wnt pathway located in the nuclear, which is associated with positive regulation of proliferation [31]. Wnt7a is a glycoprotein in the Wnt signaling pathway that is involved in the development of the anterior–posterior axis in the female reproductive tract, and plays a critical role in uterine smooth muscle patterning and the maintenance of adult uterine function [32,33].

In this study, we used the human type I endometrial cancer cell line RL95-2 (endometrioid cancer, ER, positive) and type II endometrial cancer cell line KLE (ER, negative) as cell models to study the effect of NOMAC on the growth of human endometrial cancer. We also investigated the anticancer effects of NOMAC in vivo using RL95-2 xenograft nude mice. In addition, we performed whole-genome cDNA microarray analysis to identify the effects of NOMAC on gene expression profiles in RL95-2 cells. We detected the gene expression with significant differences of SUFU and Wnt7a in RL95-2 cells, KLE cells, and RL95-2 xenografts tumor tissues by RT-qPCR and Western blotting. Our results collectively indicate that NOMAC exhibits strong anti-cancer activity both in vitro and in vivo, and it might related to the upregulation of SUFU and Wnt7a. NOMAC is a promising treatment option for patients with type I endometrial cancer, especially those with well-differentiated, endometrioid adenocarcinoma who wish to preserve their uteri or become pregnant.

## 2. Results

### 2.1. NOMAC Inhibited Cell Viability in a Dose-Dependent Manner

**Figure 1:**
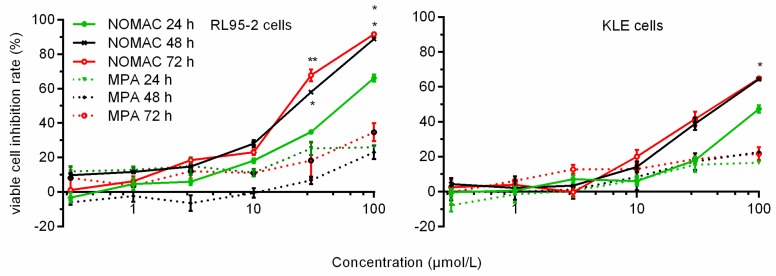
Effect of NOMAC and MPA on the viability of human endometrial cancer cells (RL95-2 and KLE) determined using the CCK-8 assay. The results are presented as the percentage of viable cells inhibited. Cells were incubated with a concentration gradient (0, 0.3, 1, 3, 10, 30, and 100 μmol/L) of NOMAC and MPA for 24, 48, and 72 h. The results are presented as the mean ± SD from three separate experiments with triplet repeat of each data. * *p* < 0.05, ** *p* < 0.01 vs. cells treated for 24 h.

The effects of NOMAC and MPA on the number of viable cultured human endometrial cancer cells (RL95-2 cells and KLE cells) were determined using the CCK-8 assay. NOMAC inhibited the growth of RL95-2 cells in a concentration-dependent manner (Figure 1). The most strong inhibitory activity was observed in the cell line RL95-2 with IC_50_ values of 19.88 μmol/L (95% confidence interval (CI): 12.01–32.91 μmol/L) at 48 h, we also observed the IC_50_ values of 21.62 μmol/L (95% CI: 12.62–36.17 μmol/L) at 72 h and 52.80 μmol/L (95% CI: 35.85–77.77 μmol/L) at 24 h (Table 1). In comparison, MPA did not inhibit the proliferation of RL95-2 cells in all tested concentrations and time points.

Regarding KLE cells, we did not find strong inhibitory activity of NOMAC and MPA. The inhibition rates of 100 μmol/L NOMAC were 33.54 ± 0.72%, 45.57 ± 0.34%, and 52.15 ± 0.52% at 24, 48, and 72 h, respectively. Its corresponding IC_50_ values were more than 100 μmol/L at 72 h. All of the MPA inhibition rate were lower than 50% (the inhibition rates of 100 μmol/L MPA are 17.50 ± 0.45%, 28.14 ± 0.17%, and 26.50 ± 1.14% at 24, 48, and 72 h, respectively (Figure 1)—hence, no corresponding IC_50_ values were calculated out. Therefore, we used a NOMAC concentration of 20 μmol/L, and an exposure period of 48 h in the following studies.

### 2.2. Effect of NOMAC on Cell Proliferation of RL95-2 and KLE

**Figure 2:**
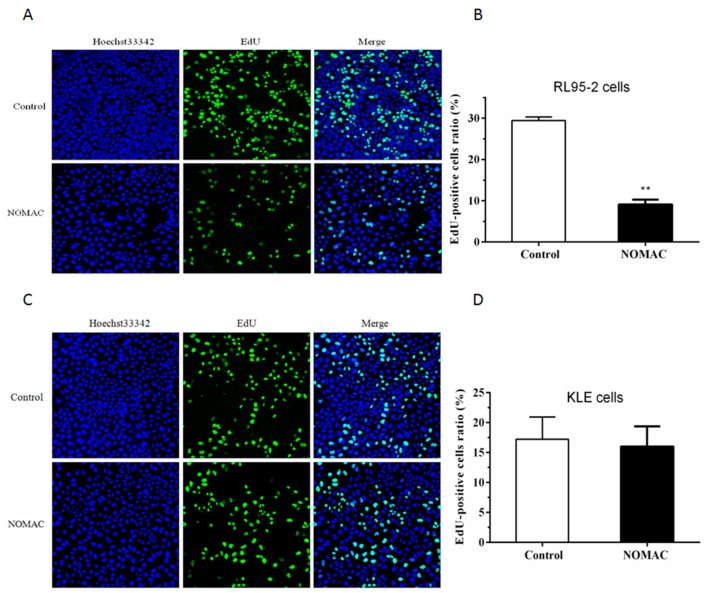
Effects of NOMAC on the cell lines of RL95-2 and KLE. All cell nuclei show blue fluorescence indicative of Hoechst33342 staining. EdU-labeled cells (green fluorescence) indicate new DNA synthesis following NOMAC (20 μmol/L) or DMSO treatment for 48 h (original magnification, 200×). All of the results that come from three independent experiments and are expressed as mean ± SD. (**A**,**B**) The relative ratio of EdU-positive RL95-2 cells is lower in the NOMAC group than in the control group at 48 h (*p* < 0.01); (**C**,**D**) the relative ratio of EdU-positive KLE cells is slightly low in the NOMAC group than the control group (*p* > 0.05). These results were typical of three independent experiments. ** *p* < 0.01 vs. control.

To further examine the effect of NOMAC on the proliferation of RL95-2 and KLE cell lines, the EdU-incorporation assay was performed to measure the inhibition of cells proliferation following treatment with NOMAC (20 μmol/L) for 48 h (Figure 2). In the RL95-2 cells, the positive ratio of EdU staining was 9.11 ± 1.16% after treatment with NOMAC and significantly lower than that in the control group (29.44 ± 0.88%, *p* < 0.01; Figure 2A,B).

In the KLE cells, the positive ratio of EdU staining was 15.55 ± 9.42% after treatment with NOMAC. There was no significant difference observed between the NOMAC treated group and the control group (17.71 ± 7.18%, *p* > 0.05; Figure 2C,D).

### 2.3. Effect of NOMAC on cDNA Microarray

To identify the underlying effect of NOMAC (20 μmol/L) on the genes expression, we conducted cDNA microarray analysis. Since no significant response to NOMAC was observed in the cell line of KLE, we therefore selected the cell line of RL95-2 to perform the experiment. By using a *p*-value cutoff of 0.05 and a fold-change cutoff of 2.0, we identified 329 genes that were upregulated, and 400 genes that were downregulated compared with the control cells. Surprisingly, we found that expression of apoptosis-related genes, such as *caspases*, *TNF*, *Bcl-2*, and *p53* did not change at all. Interestingly, we found that tumor cell proliferation-related genes of *Wnt7a*, *SUFU*, *PTCH2*, and *Gli2* changed significantly. Therefore, these genes were selected for further validation by quantitative reverse transcription polymerase chain reaction (RT-qPCR). A partial list of the differentially expressed genes is shown in Table 2.

### 2.4. Effects of NOMAC on the mRNA Levels of SUFU and Wnt7a in RL95-2 and KLE Cells

On the basis of their functions in cell proliferation, five genes of *Wnt7a*, *β-catenin*, *SUFU*, *PTCH2*, and *Gli2* from the microarray test result were chosen for further analysis. We investigated the effect of NOMAC on the mRNA expression levels of Wnt7a, β-catenin, SUFU, PTCH2, and Gli2 to identify the pathway that mediated the effects of NOMAC on cell proliferation. 

**Figure 3:**
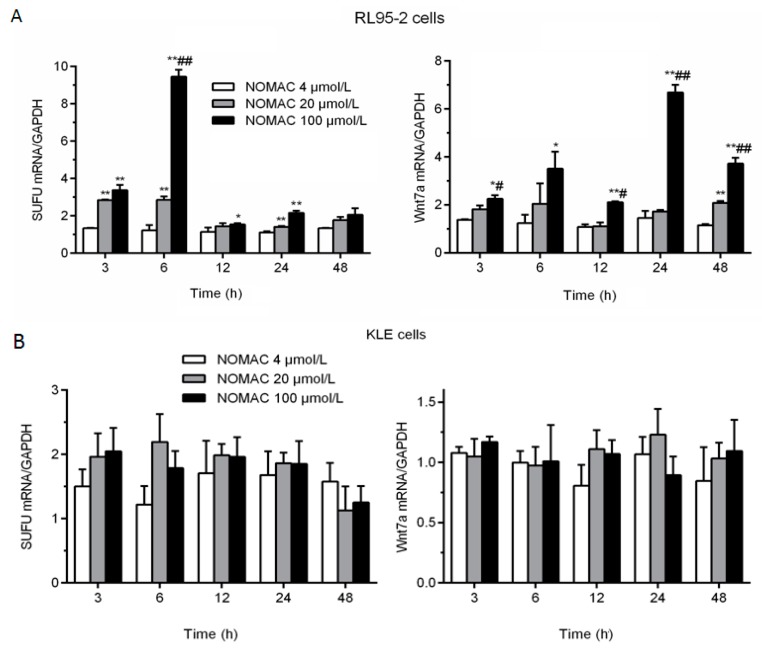
Effects of NOMAC on the mRNA levels of SUFU and Wnt7a in cultured cells (RL95-2 and KLE), as assessed by RT-qPCR. RL95-2 and KLE cells were exposed to NOMAC (0, 4, 20, and 100 μmol/L) for various time points. Data are presented as fold changes relative to the mRNA expression levels of GAPDH. All of the results were from three independent experiments and expressed as mean ± SD. (**A**) The relative abundance of SUFU and Wnt7a mRNA in RL95-2 cells; (**B**) The change in the mRNA expression levels of SUFU and Wnt7a in KLE cells. * *p* < 0.05, ** *p* < 0.01 relative to the 4 μmol/L NOMAC group; # *p* < 0.05, ## *p* < 0.01 relative to the 20 μmol/L NOMAC group.

We treated RL95-2 cells with 0, 4, 20, and 100 μmol/L NOMAC for 3, 6, 12, 24, and 48 h. The treatment of RL95-2 cells with NOMAC increased the mRNA expression of SUFU and Wnt7a, but the mRNA expression of β-catenin, PTCH2, and Gli2 did not significantly change (data not shown). SUFU expression was significantly higher in the 20 and 100 μmol/L NOMAC groups than that in the 4 μmol/L NOMAC group (*p* < 0.01) at 3, 6, and 24 h (Figure 3A). In addition, Wnt7a was strongly expressed and its level was significantly higher in the RL95-2 cells treated with 100 μmol/L NOMAC compared with that treated with 20 or 4 μmol/L NOMAC (*p* < 0.05, Figure 3A).

Furthermore, we confirmed the expression of SUFU and Wnt7a in KLE cells as well. As shown in Figure 3B, the mRNA expression levels of SUFU and Wnt7a were not significantly altered by NOMAC treatment for 3, 6, 12, 24, and 48 h.

### 2.5. Effects of NOMAC on the Protein Levels of SUFU and Wnt7a in RL95-2 and KLE Cells

**Figure 4:**
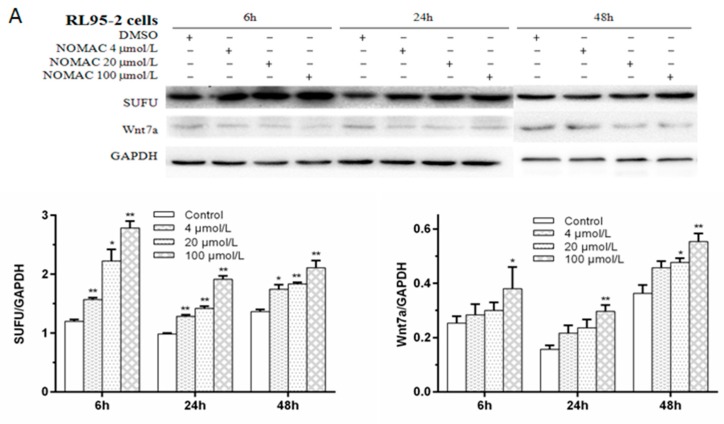

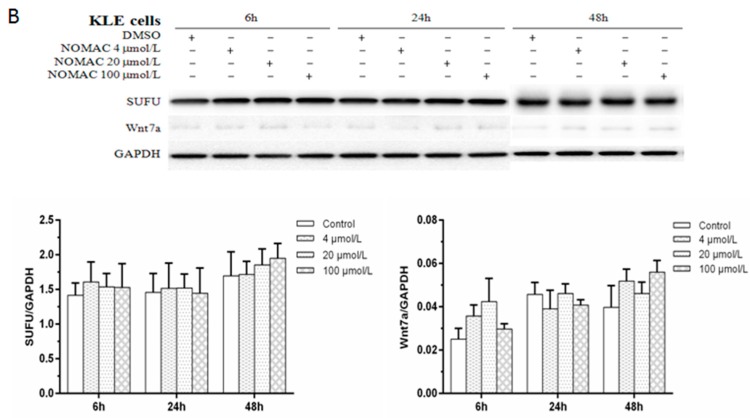
The effects of NOMAC on the protein levels of SUFU and Wnt7a in cultured cells (RL95-2 and KLE), as assessed by Western blotting. The relative amounts of SUFU and Wnt7a, collected from NOMAC-treated RL95-2 and KLE cells, were determined by Western blotting for the indicated times. GAPDH was used as a loading control. Cells were cultured with DMSO (control) or graded concentrations of NOMAC (4, 20, and 100 μmol/L) for 6, 24, and 48 h. All of the results came from three independent experiments. The data were expressed as mean ± SD. (**A**) The protein expression of SUFU and Wnt7a in response to NOMAC treatment in RL95-2 cells; (**B**) NOMAC did not alter the expression levels of SUFU and Wnt7a as assessed by Western blotting analysis in KLE Cells. * *p* < 0.05, ** *p* < 0.01 relative to the control group.

Since our studies showed that NOMAC significantly upregulate the mRNA expression levels of SUFU and Wnt7a in the RL95-2 cells but not in the KLE cells, the effect of NOMAC on the protein levels of SUFU and Wnt7a was further analyzed using Western blotting (Figure 4A). In consistent with the mRNA results, the results of Western blotting showed that NOMAC increased the protein levels of SUFU and Wnt7a (relative to GAPDH expression) in a concentration-dependent manner in RL95-2 cells. Both of the ratios of the SUFU/GAPDH and the Wnt7a/GAPDH were significantly higher in cells treated with 100 μmol/L NOMAC, compared with which treated with 4 or 20 μmol/L NOMAC (*p* < 0.05; Figure 4A).

Furthermore, we performed Western blotting assays to confirm the protein expression levels of SUFU and Wnt7a in the KLE cells. Similar to the findings in the mRNA assay, NOMAC did not alter the protein levels of SUFU and Wnt7a (Figure 4B). 

### 2.6. NOMAC Inhibits Tumor Growth In Vivo

A xenograft nude mouse model was used to test the antitumor effects of NOMAC in vivo. Forty mice were inoculated with RL95-2 cells. Three weeks later, the animals whose volume of xenograft tumor reached 100 mm^3^ were randomly divided into five groups (*n* = 8) and be administered with solvent, MPA 100 mg/kg, or NOMAC 50, 100 and 200 mg/kg, respectively. Compared to the solvent treated model animals, there were no significant changes in appearance and body weight observed in the NOMAC treated animals during our study.

**Figure 5:**
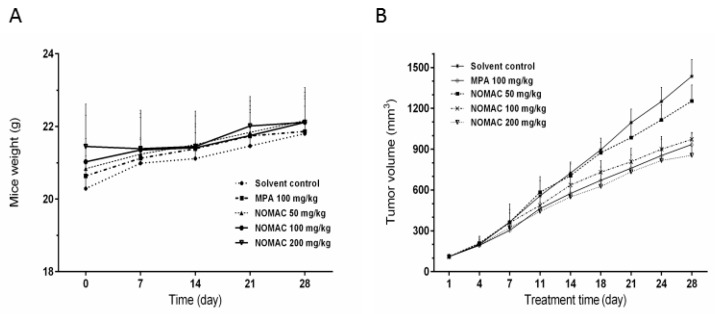

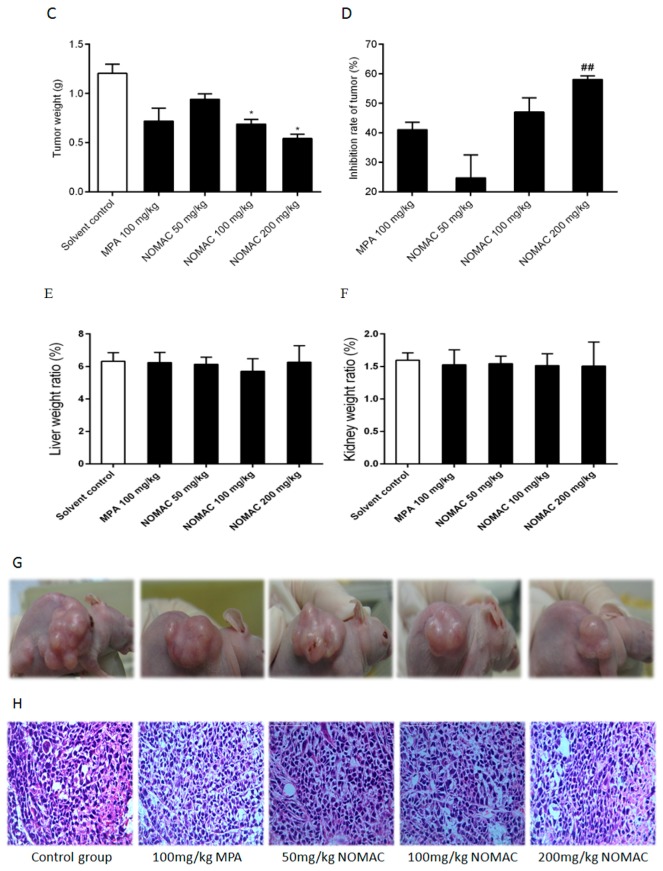
NOMAC and MPA reduced the growth of RL95-2 xenograft tumors in vivo. Mice were treated with 50, 100, and 200 mg/kg NOMAC and 100 mg/kg MPA (*n* = 8). (**A**) Body weights of mice before and during the treatment of the tumor xenografts; (**B**) Changes in tumor volume during the treatment; (**C**) Tumor weights in the mice at the end of the treatment; (**D**) The tumor growth inhibition rate. Each point represents the mean ± SD (*n* = 8); (**E**,**F**) Relative liver and kidney weights at the end of the treatment. * *p* < 0.05 vs. control, ## *p* < 0.01 vs. the 100 mg/kg MPA group; (**G**) The tumor of RL95-2 xenograft mice at the end of the experiment; (**H**) Hematoxylin and eosin staining of tumor specimens harvested from in vivo experiments (magnification, 400×).

After treatment for 28 days, tumors were harvested. The body weights and tumor weight of all animals were weighed. The body weights of the mice treated with NOMAC and MPA were similar to those of the controls (Figure 5A). However, the tumor weights in the NOMAC- and MPA (positive control)-treated groups were significantly lower than those in the solvent control mice (*p* < 0.05; Figure 5C). The relative liver and kidney weights did not differ among all the groups (Figure 5E,F). In all four treatment groups, the tumor volume decreased during the observation period (Figure 5B).

**Figure 6:**
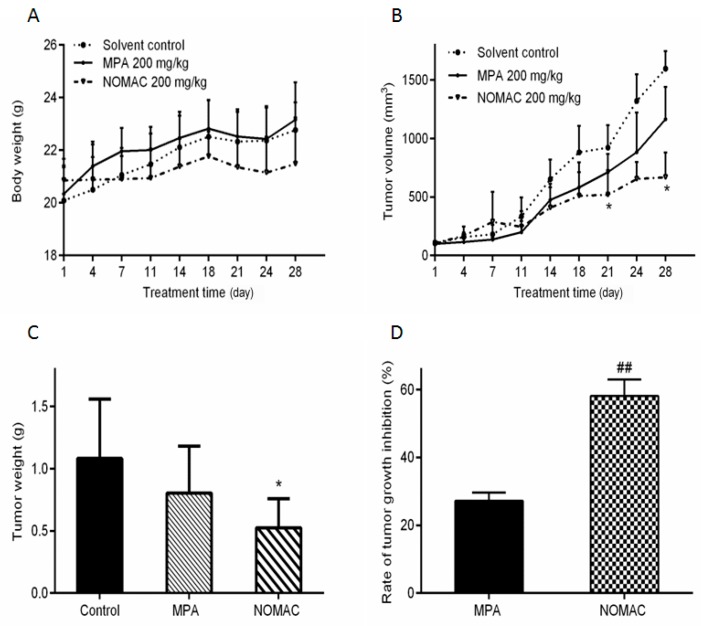
Effects of the same dose (200 mg/kg) of NOMAC and MPA on the growth of RL95-2 xenograft tumors in vivo (*n* = 8). (**A**) Body weights of mice during the treatment of the tumor xenografts; (**B**) Changes in tumor volume during the treatment; (**C**) Tumor weights at the end of treatment; (**D**) Rate of tumor growth inhibition. Each point represents the mean ± SD (*n* = 8). * *p* < 0.05 vs. control; ## *p* < 0.01 vs. the MPA group.

From Figure 5D, we found that the tumor inhibition in the 200 mg/kg NOMAC treatment group was significantly lower than that in the MPA treatment group (*p* < 0.01), but no remarkable difference was observed in the tumor inhibition rate between the 100 mg/kg NOMAC treatment group and the 100 mg/kg MPA treatment group (*p* > 0.05), so we further compared the efficacy of NOMAC and MPA at the same dose of 200 mg/kg. The results demonstrated that 200 mg/kg NOMAC (58.13 ± 6.53%) induced a significantly stronger tumor growth inhibition than 200 mg/kg MPA (27.01 ± 2.13%, *p* < 0.01; Figure 6D). 

Hematoxylin and eosin staining showed that the tumors in the mice had the same biological characteristics as endometrioid adenocarcinoma in the human endometrium (Figure 5G,H). This result suggests that the mouse model we used in this study reflects the clinicopathogical characteristics observed in human type I endometrial cancer.

### 2.7. NOMAC Suppresses the Expression of SUFU and Wnt7a in Tumor Tissues

**Figure 7:**
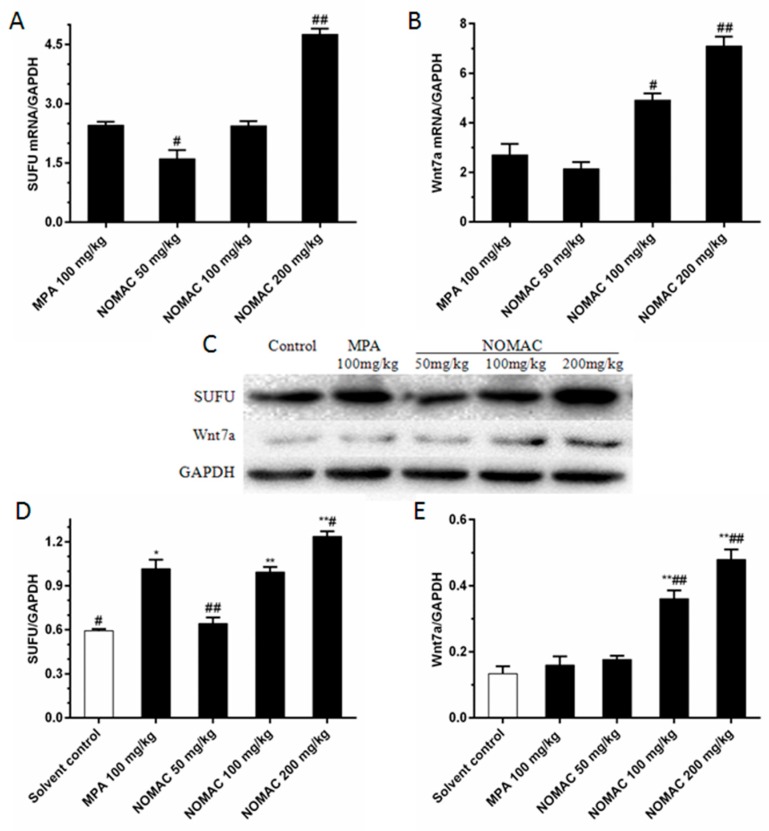
Effects of NOMAC and MPA on the levels of SUFU and Wnt7a in xenograft mouse tumor tissues, as assessed by RT-qPCR and Western blotting. Mice were treated with 50, 100, or 200 mg/kg NOMAC or 100 mg/kg MPA. (**A**,**B**) The change in mRNA expression levels of SUFU and Wnt7a in RL95-2 xenograft mice tumor tissues. Data are presented as fold changes relative to the expression of GAPDH. * *p* < 0.05, ** *p* < 0.01 vs. control; # *p* < 0.05, ## *p* < 0.01 vs. MPA; (**C**) The protein expression of SUFU and Wnt7a in response to NOMAC and MPA treatment in xenograft mouse tumor tissues; (**D**,**E**) SUFU and Wnt7a protein production is determined using Western blot. GAPDH is used as a loading control. * *p* < 0.05, ** *p* < 0.01 vs. relative to the control group; # *p* < 0.05, ## *p* < 0.01 vs. relative to the MPA group.

Moreover, we evaluated the expression levels of *SUFU* and *Wnt7a* in tumor tissues in the mice by using RT-qPCR. We found that *SUFU* expression increased in the NOMAC treated mice in a concentration-dependent manner (Figure 7A). Compared to the 100 mg/kg MPA group, there was a pronounced increase in *SUFU* expression in the group treated with 200 mg/kg NOMAC (*p* < 0.01). The mRNA expression of Wnt7a was upregulated in the NOMAC-treated group in a concentration-dependent manner compared to that in the solvent control group. Compared to the 100 mg/kg MPA group, the Wnt7a mRNA level was significantly increased in the 100 mg/kg NOMAC group (*p* < 0.05) and 200 mg/kg NOMAC group (*p* < 0.01; Figure 7B).

In consistent with the mRNA levels, Western blotting analysis demonstrated that both of SUFU and Wnt7a expression were significantly upregulated in tumor tissues with NOMAC treatment (*p* < 0.01; Figure 7C–E). Compared with the solvent control, there was an obvious increase in the SUFU/GAPDH protein levels in the 100 mg/kg MPA (*p* < 0.05) and 100 and 200 mg/kg NOMAC (*p* < 0.01) treated groups. 100 and 200 mg/kg NOMAC treatment significantly increased the protein level of Wnt7a compared to that of the 100 mg/kg MPA group (*p* < 0.01) and the control group (*p* < 0.01).

## 3. Discussion

In the present study, we found that NOMAC significantly suppressed the growth of the human type I endometrial cancer cell line RL95-2, but not in the KLE, the type II endometrial cancer cell line in vitro. Furthermore, the anticancer activity of NOMAC was validated by using an RL95-2 xenograft nude mouse model. Our data indicate that NOMAC has a stronger inhibitory effect on the progression and development of these xenograft tumors than MPA. In addition, we revealed that SUFU and Wnt7a expression were upregulated in response to NOMAC treatment in vitro and in vivo.

At the first beginning, we detected the expression levels of ER-α and PR in RL95-2 and KLE cells by Western blotting. The results showed that RL95-2 cells expressed ER-α and PR, but KLE cells did not express ER-α or PR, which is consistent with other reports [34,35,36]. We confirmed that the RL95-2 cell we used in the experiment belonged to a type I hormone-dependent endometrial cancer cell line, and the KLE cell was a type II non-hormone-dependent endometrial cancer cell line (data not shown). Then we further utilized the two cell lines to evaluate the effects of NOMAC on the growth of human endometrial cancer cells.

By analyzing the inhibitory rates and IC_50_ values, we showed that NOMAC significantly inhibited RL95-2 cell growth in vitro. However, as for a type II endometrial cancer cell line, we found that NOMAC had little inhibition on the growth of KLE cells even when the concentration is up to 100 µmol/L. The KLE cell line with defective ER is derived from poorly differentiated cancer, and a study reported that KLE is not hormone-sensitive [34]; while RL95-2 cells are derived from a moderately differentiated adenosquamous carcinoma of the endometrium [36,37], and sensitive to the treatment of progesterones. Our result suggests that NOMAC exhibited stronger inhibitory effect on the growth of hormone-sensitive type I endometrial cancer cells than that of hormone-insensitive type II endometrial cancer cells. With the EdU experiments, we further confirmed that NOMAC had a significant inhibitory effect on the DNA synthesis of RL95-2 cells, but not on KLE cells. Taken together, the results indicate that NOMAC might have a better therapeutic effect on type I endometrial cancer than on type II endometrial cancer, which could explain the reason why progestin can be used for the fertility preserving treatment in young women with early endometrial cancer [38,39]. We therefore used type I endometrial cancer cell line of RL95-2 cells in subsequent experiments.

We further confirmed our findings in vivo with a xenograft nude mouse model. We inoculated 40 nude mice with RL95-2 cells. Approximately 20 days later, the tumor xenograft were observed in all inoculated mice, and the volume of the xenograft were up to 100 mm^3^. Clinically, MPA is administered orally at the dose of 500 mg in the treatment of human endometrial cancer. This dose is equal to 102.75 mg/kg for a mouse, we therefore used the dose of 100 mg/kg of MPA to treat the nude mice; Moreover, we designed the dose of NOMAC with 50, 100, and 200 mg/kg in our study. We found that the tumor size in the mice treated with NOMAC and MPA was smaller than that in the control mice, but in all nude mice with RL95-2 xenografts, we did not observe significant changes in body weight and abnormality in the liver and kidney after the animal were anatomy, and there was no significant difference in the relative liver and kidney weights at the end of the treatment in any group. These results indicate that no serious adverse effects on nude mice were appeared among all NOMAC and MPA treatment groups. We also found that the tumor growth inhibitory effect of NOMAC increased in a concentration-dependent manner, and it was stronger than MPA. In addition, hematoxylin and eosin staining showed that the characteristics of the neoplastic endometrial cancer which was developed in the mice were similar to those of endometrioid adenocarcinoma found in humans. These results show that the therapeutic effect of NOMAC could be better than MPA in the treatment of type I endometrial cancer.

Furthermore, we performed whole-genome analysis and confirmed the existence of differences of multiple genes in the NOMAC treated RL95-2 cells compared with that of control cells. Surprisingly, no apoptosis-related genes, including *caspases*, *TNF*, *Bcl-2*, *p53* and etc. were found to change significantly. Interestingly, the genes of *SUFU*, *Gli2*, *Wnt7a*, and *PTCH2* closely related to tumor cell proliferation were significantly upregulated. Then, we detected the mRNA expression levels of these genes (*Wnt7a*, *β-catenin*, *SUFU*, *Gli2*, and *PTCH2*) by RT-qPCR to validate the microarray results after NOMAC (0, 4, 20, and 100 μmol/L) treatment for 3, 6, 12, 24 and 48 h. Consistent with the microarray data, RT-qPCR analyses showed that SUFU and Wnt7a were expressed at significantly higher levels in NOMAC-treated RL95-2 cells than in control cells at 6, 24, and 48 h. To further confirm the expression of SUFU and Wnt7a in RL95-2 cells, we detect the protein expression levels by Western blotting. In consistent with our RT-qPCR results, NOMAC significantly increased the protein expression of SUFU and Wnt7a. This result suggests that inhibition of cell proliferation by NOMAC might be involved in SUFU and Wnt7a in the RL95-2 cell line. The effect of NOMAC on the expression of SUFU and Wnt7a were performed by RT-qPCR and Western blotting in KLE cells as well. However, the results showed that NOMAC could not alter the mRNA and protein expression levels of SUFU and Wnt7a in KLE cells. By using RT-qPCR and Western blotting analyses we found that SUFU and Wnt7a were upregulated in a dose-dependent manner in RL95-2 tumor xenograft tissues after NOMAC treatment, which is consistent with the results in vitro, Taken together, these results show that NOMAC inhibited the proliferation of type I endometrial cancer cell RL95-2 possibly associate with upregulating SUFU and Wnt7a in vivo and in vitro, but had no significant effect on the proliferation of type II cell line KLE and the expression of SUFU and Wnt7a.

SUFU is a key inhibitor of Hh signaling pathway, and is part of the mechanism that negatively regulates the Gli activity. By binding and inactivating Patched (Ptc/PTCH), Hh signaling pathway unleashes the Smoothened (Smo) transmembrane protein to trigger downstream intracellular events. A previous study showed that *SUFU* expression is significantly lower in simple and complex endometrial hyperplasia than that in normal endometrium [27]. Accordingly, the expression of SUFU, Gli2, and PTCH2 should be theoretically regulated by NOMAC in the endometrial cancer; however, our RT-qPCR results showed that the mRNA levels of Gli2 and PTCH2 did not change significantly as SUFU upregulation, and they were inconsistent with cDNA microarray analysis. It suggests that we should further study the roles of Gli2 and PTCH2 in the effect of NOMAC inhibiting the growth of RL95-2 cells.

In the present study, we also investigated the expression of β-catenin and Wnt7a, which belong to the Wnt pathway, to determine the expression changes in the endometrial cancer cell line of RL95-2. Unfortunately, we did not find that *β-catenin* showed a distinct alteration in the cDNA microarray analysis and in quantification of mRNA. We therefore focused on investigating the alteration of Wnt7a, in which its role in proliferation was explored [29,30]. Previous studies suggest that Wnt7a is a steroidal regulated gene, which can be upregulated by progestogens [31]. In this study, we found that NOMAC significantly increased expression of Wnt7a and suppressed RL95-2 cell proliferation. Ramos-Solano et al. reported that restorating Wnt7a expression decreased HaCa cell proliferation and silencing Wnt7a induced an increased proliferation in HaCaT cells [40]. As a result, we presume that Wnt7a may be involved in the anti-proliferative effect of NOMAC on RL95-2 cells. Taken together, these results suggest that NOMAC suppressing the growth of RL95-2 cell may relate to altering the expression of the key genes of the Wnt and Hedgehog signaling pathways, such as Wnt7a and SUFU, but did not alter the expression of β-catenin, Gli2, and PTCH2 in the above signaling pathways. However, NOMAC did not significantly inhibit the growth of the type II endometrial cancer cell line of KLE, and did not significantly upregulate the expression of SUFU and Wnt7a in the KLE cells. Taken together, the upregulation of SUFU and Wnt7a may be associated with the anticancer effects of NOMAC inhibiting RL95-2 cell proliferation. To our knowledge, this is the first study to report increased SUFU and Wnt7a levels may associated with the proliferation of type I endometrial cancer cells.

In our future study, we intend to confirm our results in human tumor tissues, and we will further investigate the mechanism underlying the antitumor action of NOMAC by means of SUFU and Wnt7a gene knockdown in RL95-2 cells. We will further study the relationship between the antitumor effect of NOMAC and Hh or Wnt signaling pathways.

## 4. Materials and Methods

### 4.1. Chemicals and Reagents

NOMAC (C_23_H_30_O_4_; molecular weight, 370.48; purity, >98%) was obtained from Lijiang Yinghua Bio-Pharmaceutical Co., Ltd. (Kunming, China). MPA was obtained from Xianju Pharma (Ningbo, China). Dimethyl sulfoxide (DMSO) was obtained from Sigma Chemical Co. (St. Louis, MO, USA). Sodium carboxymethylcellulose (CMC-Na) and Tween-80 were purchased from Sinopharm Chemical Reagent Co. Ltd. (Shanghai, China). NOMAC and MPA were dissolved in DMSO, which was used at a final concentration of <0.1% in the culture medium.

TRIzol reagent and RIPA lysis buffer were purchased from Invitrogen Co. (Carlsbad, CA, USA). Protease Inhibitor Cocktail was purchased from Thermo Scientific (Rockford, IL, USA). RNeasy Plus Mini Kits, QuantiTect Reverse Transcription Kits, and QuantiNova SYBR Green PCR Kits were purchased from Qiagen (Dusseldorf, Germany). Rabbit monoclonal antibody against SUFU (C81H7; 54 kDa) and rabbit polyclonal antibody against GAPDH (D16H11; 37 kDa) were purchased from Cell Signaling Technology (Beverly, MA, USA). Wnt7a (ab100792; 41 kDa) rabbit polyclonal antibody were purchased from Abcam Biotechnology (Cambridge, UK). Bicinchoninic acid Protein Assay Kits (BCA, P0012S) were purchased from Beyotime Biotechnology (Shanghai, China), and enhanced chemiluminescence (ECL) detection kits were purchased from Thermo Fisher Scientific Inc. (Waltham, MA, USA).

### 4.2. Cell Culture

The RL95-2 cell line was obtained from American Type Culture Collection (ATCC). The KLE cell line was obtained from China Center for Type Culture Collection. The human endometrial cancer cell lines RL95-2 and KLE cells were cultured in Dulbecco modified Eagle medium/F12 (Thermo Fisher Scientific) containing 10% (*v*/*v*) heat-inactivated fetal bovine serum (FBS; Thermo Fisher Scientific) at 37 °C in a humidified atmosphere containing 5% CO_2_.

### 4.3. Cell Viability Assay

Cells were cultured in 96-well plates at a density of 8000 cells/well and treated with NOMAC (0.3, 1, 3, 10, 30, or 100 μmol/L) or DMSO for 24, 48, and 72 h. Cell proliferation was determined using the CCK-8 Assay Kit (Dojindo Molecular Technologies, Kumamoto, Japan). We added 10 μL of CCK-8 solution to each well and incubated the cells at 37 °C for 2 h. The absorbance (optical density) was read with a spectrophotometric plate reader (Bio-Tek ELX-800, Winooski, VT, USA) at 450 nm to determine cell viability [41]. The results are presented as the percentage of cell growth inhibition (%), the half-maximal inhibitory concentration (IC_50_), and the corresponding 95% CI [41]. The cell growth inhibition rate (%) was calculated using the following equation: (1 − OD of NOMAC-treated wells/OD of control wells) × 100% [42]. The IC_50_ and its corresponding 95% CI were calculated from the dose–response curve using GraphPad Prism 6.02 (GraphPad Software Inc., San Diego, CA, USA).

### 4.4. EdU Cell Proliferation Assay

The effect of NOMAC on cell proliferation was also determined using the EdU (5-ethynyl-2′-deoxyuridine) incorporation assay using an EdU Assay Kit (RiboBio, Guangzhou, China). In brief, cells (6 × 10^4^ cells/well) were cultured in triplicate in 3.5 mm glass Petri dishes and transfected with NOMAC (20 μmol/L) or DMSO for 48 h. Then, cells were incubated with 50 μmol/L EdU for 2 h at 37 °C. At room temperature, the cells were fixed with 4% paraformaldehyde for 30 min and treated with 0.5% Triton X-100 for 30 min to permeabilize them [43]. After being washed with phosphate-buffered saline (PBS) three times, the cells were incubated with 1× Apollo reaction cocktail (500 μL/well) for 30 min. Cell DNA was stained with 10 μg/mL Hoechst 33342 (500 μL/well) for 30 min and then visualized using fluorescence microscopy [43]. Ten groups of confluent cells were randomly selected from each sample image. EdU-positive cells in the fluorescent image were counted using Nikon A1 laser confocal microscopy (Nikon Instruments Inc., Shanghai, China), and the relative positive ratio was calculated from the average value of the 10 groups [43]. Image lab software (version 4.0, Bio-Rad laboratories, Inc., Hercules, CA, USA) was used to count the number of EdU-labeled cells and Hoechst 33342-labeled cells.

### 4.5. Microarray Analysis

Total RNAs from RL95-2 cells treated with 20 μmol/L NOMAC or DMSO for 48 h were extracted using RNeasy Plus Mini Kit (Qiagen). RNA quantity and quality were measured using Nanodrop 2000 (Thermo Scientific, Jessup, MD, USA) [42]. RNA integrity was assessed using standard denaturing 2% agarose gel electrophoresis [42]. The human gene expression 4× 44 K v2 Microarray (Agilent Technologies, Santa Clara, CA, USA) was used for the experiments. Sample labeling and array hybridization were performed according to the Agilent One-Color Microarray-Based Gene Expression Analysis protocol (Arraystar Inc., Rockville, MD, USA) [44]. Labeled cRNA (1 μg) was fragmented by adding a 10× Blocking Agent (11 μL) and a 25× Fragmentation Buffer (2.2 μL); it was then heated at 60 °C for 30 min, and finally, the labeled cRNA was diluted with a 2× GE Hybridization buffer (55 μL). Next, hybridization solution (100 μL) was dispensed into the gasket slide and assembled to the gene expression microarray slide. In an Agilent Hybridization Oven, the slides were incubated for 17 h at 65 °C. The hybridized arrays were washed, fixed, and scanned using the Agilent Microarray Scanner (Agilent model number: G2565BA) [41]. Agilent Feature Extraction software (version 11.0.1.1) was used to analyze the acquired array images [43]. Quantile normalization and subsequent data processing were performed using the GeneSpring GX v12.1 software (Agilent Technologies) [43]. After quantile normalization of the raw data, genes that were expressed in at least three of the six samples were chosen for further analysis [43]. Differentially expressed genes with statistical significance were identified through fold changes. Hierarchical clustering was performed using the Agilent GeneSpring GX software (version 12.1). Gene ontology analysis and Kyoto Encyclopedia of Genes and Genomes pathway analysis were performed using the standard enrichment computation method with differentially expressed data obtained with a cutoff value of a 2-fold change [43].

### 4.6. RT-qPCR Analysis

Total RNA was extracted from cultured KLE cells, RL95-2 cells and RL95-2 xenograft tumor tissues using RNeasy Plus Mini Kits (Qiagen), according to the manufacturer’s protocol. RNA was qualified using Nanodrop 2000, and its integrity was determined using 2% (*w*/*v*) agarose gel electrophoresis. Samples of 1 μg total RNA from each sample were reverse transcribed to cDNA by using the QuantiTect Reverse Transcription Kit (Qiagen). The cDNA was amplified using qPCR with the following specific primers: GAPDH, 5′-GGGAAACTGTGGCGTGAT-3′ (forward primer), and 5′-GAGTGGGTGTCGCTGTTGA-3′ (reverse primer); SUFU, 5′-CCTCCAGATCGTTGGTGTCT-3′ (forward primer) and 5′-TCCGCATGTCAGTTATCAGC-3′ (reverse primer); and Wnt7a, 5′-TGCCCGGACTCTCATGAAC-3′ (forward primer) and 5′-GTGTGGTCCAGCACGTCTTG-3′ (reverse primer). We performed qPCR with the QuantiNova SYBR Green PCR Kit (Qiagen) and the LightCycler480 (Roche Applied Science, Penzberg, Germany) real-time PCR system. The PCR conditions were 10 μL of SYBR Green PCR Master Mix, 2 μL of each of forward and reverse primer (0.7 μmol/L), and 2 μL of sample; ddH_2_O was added to obtain a final volume of 20 µL. PCR was performed using an initial denaturation step for 5 min at 95 °C, 40 cycles of amplification at 95 °C for 10 s, and 59 °C for 20 s, and an extension at 72 °C for 20 s. A melting curve was obtained at the end of each run, and the PCR products were analyzed on 2% (*w*/*v*) agarose gels to discriminate specific from nonspecific cDNA products. The data were quantified using the 2^−ΔΔ*C*p^ method, and the relative fold changes were corrected according to the level of GAPDH expression.

### 4.7. Western Blot Analysis

RL95-2 and KLE cells treated with NOMAC (4, 20, or 100 μmol/L) or DMSO and RL95-2 xenograft tumor tissues were lysed in an RIPA buffer supplemented with Protease Inhibitor Cocktail on ice. Clear lysates were obtained after centrifugation at 12,000× *g* for 15 min at 4 °C, and the protein was quantified using the bicinchoninic acid protein assay with bovine serum albumin as a standard. Primary antibodies against SUFU and Wnt7a were diluted to 1:1000. The primary GAPDH antibody was diluted to 1:5000, and 20 μg samples were loaded onto 12% sodium dodecyl sulfate polyacrylamide gel electrophoresis gels for electrophoresis and then transferred onto polyvinylidene difluoride membranes [43]. The membranes were blocked with 5% bovine serum albumin for 1 h at room temperature and hybridized overnight with specific primary antibodies at 4 °C [42]. The protein bands were visualized using the ECL SuperSignal West Femto Detection Kit (Thermo Scientific) after hybridization with a horseradish peroxidase-conjugated goat anti-rabbit secondary antibody (Cell Signaling Technology) [43]. The membranes were stripped with Restore Western Blot Stripping Buffer and probed with GAPDH antibody for the protein loading control. The relative levels of protein were obtained by taking the ratio of the band intensity of the target protein against that of GAPDH. Statistical analysis was performed on the average protein level of three samples. The relative levels of proteins were semi-quantitatively determined using the Image Lab gray-scale scanning software (version 4.0, Bio-Rad Laboratories, Inc.) [42].

### 4.8. Xenograft Model and Treatment

Female athymic nude mice (BALB/c; body weight, 18–20 g; age, 6–7 weeks) were purchased from Sino-British Experiment Animals (Shanghai, China). Mice were housed under specific-pathogen-free conditions in a laminar air-flow cabinet maintained at 25 °C with 50 ± 10% humidity and a 12 h dark/light cycle [43]. Mice had free access to water and food throughout the studies. Each animal was weighed once per week. All surgical and experimental procedures were reviewed and approved by the Laboratory Animal Ethics Committee at the Shanghai Institute of Planned Parenthood Research (approval no., 2015-05, 21 May 2015), and the welfare of the mice received the highest consideration during the study. All mice were allowed to acclimate to their environment for 1 week prior to the experiments and were fasted overnight on the day before the experiments. Logarithmic phase RL95-2 cells (1 × 10^7^ cells/mL, 0.2 mL/mouse) were subcutaneously injected into the mice on the right flank. Tumorigenicity was determined by assessing the tumor volume over time. All mice developed single palpable tumors within 2 weeks after inoculation. Tumors were serially measured every Tuesday and Friday using a digital caliper, and the tumor volume was calculated using the following formula: length × width × width × 0.5. Treatment was initiated when the tumor reached about 100 mm^3^ in volume, and this day was designated Day 1. On Day 1, mice were randomly split into five groups with eight mice in each group and received the following interventions: solvent control, 100 mg/kg MPA, and 50, 100, or 200 mg/kg NOMAC. MPA and NOMAC were dissolved in normal saline solution with 0.5% CMC-Na and 0.1% Tween-80. Each mouse was administered its designated treatment via intragastric gavage once daily for 28 days. To compare the efficacy of MPA and NOMAC at the same dose, we randomly divided another 24 xenograft mice into solvent control, NOMAC (200 mg/kg), and MPA (200 mg/kg) groups, and subjected them to the same protocols. Animals were sacrificed by anesthesia 1 day after the last treatment, and tumors were harvested. One fifth of the tumors were fixed in 4% paraformaldehyde, and the rest were rapidly frozen with liquid nitrogen and then stored at −80 °C.

### 4.9. Hematoxylin and Eosin Staining

Mice were anesthetized and then dissected. The xenograft tumor tissues were taken out and embedded in paraffin, sliced into 4-µm-thick sections, and stained with hematoxylin and eosin for histological examination under a light microscope.

### 4.10. Statistical Analysis

In this paper we define all of the error bars as standard deviation. Data are presented as means ± standard deviation. Statistical significance was determined using one-way analysis of variance and the least significant differences test (SPSS 17.0 software; SPSS Inc., Chicago, IL, USA). Differences with *P*-values of less than 0.05 were considered statistically significant. All graphs were generated using GraphPad Prism 6.02 (GraphPad Software Inc., San Diego, CA, USA).

## 5. Conclusions

In summary, at the tested concentrations, the mechanism of action of NOMAC may be involved in the regulation of the activity of SUFU and Wnt7a in type I endometrial cancer cell line RL95-2 and RL95-2 xenograft tumor tissues, but not in type II endometrial cancer cell line KLE. These results provide novel insights into understanding the molecular mechanisms and underlying the anticancer activity of NOMAC, which may facilitate the development of more efficacious and safer drugs for type I endometrial cancer, especially for young patients with strong fertility needs.

## Figures and Tables

**Table 1:** Antiproliferative activity of nomegestrol acetate (NOMAC) and medroxyprogesterone acetate (MPA) as determined by IC_50_ values (in μmol/L) for human endometrial cancer cell lines RL95-2 and KLE.

Cell Lines	Drugs	IC_5__0_ (95% CI) µmol/L		
		24 h	48 h	72 h
RL95-2	NOMAC	52.80 (35.85–77.77)	19.88 (12.01–32.91)	21.62 (12.62–36.17)
	MPA	>100 (--)	>100 (--)	>100 (--)
KLE	NOMAC	>100 (--)	>100 (--)	>100 (--)
	MPA	>100 (--)	>100 (--)	>100 (--)

The results are presented as IC_50_ (inhibits cell proliferation by 50%) and its 95% CI (confidence interval). IC_50_ was defined as the drug concentration required to reduce the number of living cells by 50% after incubation with 0.3–100 μmol/L of the drug for 24, 48, and 72 h. When the inhibition rate is less than 50% of the maximum concentration, the corresponding IC_50_ value is higher than 100 μmol/L, so we did not shown the IC_50_ and CI and replaced it by >100 (--). Values shown are from triple wells per data point obtained in three separate cultures.

**Table 2:** Differentially expressed genes in response to NOMAC treatment in human endometrial cancer RL95-2 cells, according to cDNA microarray analysis.

Gene ID	Gene Symbol	*p*-Value	Fold Change	Regulation
NM_019851	*FGF20*	0.015	5.937	Up
NM_004625	*Wnt7a*	0.017	3.086	Up
NM_016169	*SUFU*	0.023	2.738	Up
NM_005270	*GLI2*	0.024	2.072	Up
NM_001719	*BMP7*	0.028	4.676	Down
NM_003810	*TNFSF10*	0.030	3.278	Down
NM_003999	*OSMR*	0.026	3.218	Down
NM_201282	*EGFR*	0.014	2.307	Down

The resulting *p*-values were adjusted for multiple testing by the Benjamini–Hochberg adjustment. Gene ID: Genbank accession number.
